# Exploring the diversity and pathogenicity of *Talaromyces* species isolated from clinical in Southern China

**DOI:** 10.3389/fmicb.2025.1610481

**Published:** 2025-06-26

**Authors:** Liuwei Liao, Kaisu Pan, Dongyan Zheng, Shuangjie Wang, Weixuan Wu, Cunwei Cao

**Affiliations:** ^1^Department of Dermatology, The First Affiliated Hospital of Guangxi Medical University, Nanning, China; ^2^Guangxi Key Laboratory of AIDS Prevention and Treatment, Guangxi Medical University, Nanning, China; ^3^Guangxi Key Laboratory of Mycosis Prevention and Treatment, Nanning, China; ^4^Guangxi Scientific and Technological Innovation Cooperation Base of Mycosis Prevention and Control, Nanning, China; ^5^Department of Laboratory, Maternity and Child Health Care of Guangxi Zhuang Autonomous Region, Nanning, China

**Keywords:** *Talaromyces*, Southern China, diversity, drug susceptibility, pathogenicity

## Abstract

In recent years, some species of *Talaromyces* have emerged as human pathogens but rarely reported from Southern China, the endemic region of *Talaromyces marneffei*. To investigate the diversity of *Talaromyces* species in Southern China, we collected 59 clinical *Talaromyces* isolates, which were identified as 11 different species through molecular analysis. Notably, several species exhibited phenotypic characteristics similar to *T. marneffei*, such as red pigment production at 25°C and monoverticillate or biverticillate conidiophores with globose to subglobose conidia, potentially leading to misidentification. Antifungal susceptibility testing revealed distinct drug susceptibility profiles between *T. marneffei* and other species. Furthermore, seven species demonstrated growth at 37°C and induced inflammatory lung damage in mice, suggesting their pathogenic potential. These emerging *Talaromyces* pathogens were primarily isolated from respiratory tract samples of immunocompromised patients. Our findings highlight the rich diversity of *Talaromyces* species clinically in Southern China, emphasizing the critical importance of considering their potentially pathogenic.

## 1 Introduction

The genus *Talaromyces* (*Trichocomaceae*, *Eurotiales*) is widely distributed in nature and closely associated with human activities. Based on polyphasic taxonomy, it is classified into eight sections: *Bacillispori*, *Helici*, *Islandici*, *Purpurei*, *Subinflati*, *Tenues*, *Talaromyces*, and *Trachyspermi* (Yilmaz et al., 2014; Sun et al., 2020). With the advancement of molecular diagnostic techniques, the number of *Talaromyces* species has rapidly increased, exceeding 180 described species to date.

While *Talaromyces marneffei* remains a significant cause of high-mortality talaromycosis (Cao et al., 2019; Narayanasamy et al., 2021), emerging pathogenic species within the genus have been increasingly reported. For example, *T. indigoticus* has been implicated in human skin and nail infections in Panama (Weisenborn et al., 2010), and *T. kabodanensis*, *T. subaurantiacus*, *T. alveolaris*, and *T. rapidus* have been associated with respiratory tract infections (Guevara-Suarez et al., 2017). Moreover, *T. piceum* has been linked to fungemia and rib osteomyelitis in patients with X-linked chronic granulomatous disease (Horré et al., 2001; Santos et al., 2006). Notably, phenotypic similarities, such as monoverticillate or biverticillate conidiophores and red pigment production, can lead to the misidentification of other *Talaromyces* species as *T. marneffei*. A study from Indonesia demonstrated that nine clinical isolates previously identified as *T. marneffei* based on phenotype were subsequently confirmed as *T. atroroseus* through molecular analysis (Surja et al., 2020). Therefore, molecular methods are essential for accurately identifying clinical *Talaromyces* strains.

In recent years, some clinical *Talaromyces* species isolated from patients were reported in non-*T. marneffei*-endemic areas (Li et al., 2019). In this study, we have observed the emergence of *Talaromyces* species other than *T. marneffei* in clinical samples from Southern China, a region endemic to *T. marneffei*. Some of them could grow at 37°C—the temperature of the human body, suggesting a potential pathogenic ability. To investigate the potential pathogenicity of these species, we collected clinical *Talaromyces* isolates and identified them using molecular analysis. Comparative studies on morphology, drug susceptibility, and clinical features were conducted between *T. marneffei* and other *Talaromyces* species. Additionally, based on the principle of Koch’s postulate that strains could be re-isolated from the experimentally infected host (Singh et al., 2016), we assessed the pathogenicity of these *Talaromyces* species through animal models to inform potential treatment strategies.

## 2 Materials and methods

### 2.1 Isolates

Fifty-nine *Talaromyces* strains were isolated from different patients at The First Affiliated Hospital of Guangxi Medical University between September 2020 and March 2022. Briefly, clinical samples were aseptically collected and subsequently isolated on Sabouraud dextrose agar (SDA) medium at 27°C for 14 days. Pure fungal isolates were obtained through the plate streaking methods in preparation for downstream experimental analyses. All isolates were stored in the Guangxi Key Laboratory of Mycosis Research and Prevention.

### 2.2 Morphological analysis

Macroscopic characteristics were evaluated on SDA, czapek yeast autolysate agar (CYA) and malt extract agar (MEA). Isolates were cultured at 25°C and 37°C for a 14-day incubation period. Colony texture, color, soluble pigment production, and microscopic features, including conidiophore branching and conidial morphology, were recorded.

### 2.3 Phylogenetic analysis

Strains were cultured on SDA medium for 7 to 14 days prior to DNA extraction. DNA was extracted using the MightyPrep reagent (Takara, Beijing, China), and PCR amplification was performed with MightyAmp DNA Polymerase Ver.3 (Takara). The ITS, *BenA*, and *CaM* genes were amplified following protocols described in previous studies (Houbraken and Samson, 2011; Yilmaz et al., 2014). Phylogenetic analyses were conducted using the Maximum Likelihood (ML) method in MEGA v.7.0 based on combined ITS, *BenA*, and *CaM* gene sequences. ML analyses were supported by 1,000 bootstrap replicates, and bootstrap values ≥70 were considered significant.

### 2.4 Antifungal susceptibility testing

Antifungal susceptibility testing of the isolates was performed using the broth microdilution method as outlined in the Clinical and Laboratory Standards Institute (CLSI) M38-A3 standard for filamentous fungi (Wayne, 2017) and referred to previous studies (Guevara-Suarez et al., 2016; Guo et al., 2021). Briefly, strains were grown on potato dextrose agar for 7 to 10 days at 27°C until good conidiation. The conidia for each strain were resuspended in sterile 0.85% saline to a concentration of 5 × 10^6^ CFU/mL. The susceptibility profiles of itraconazole (ITC), voriconazole (VRC), posaconazole (PSC), isavuconazole (ISC), fluconazole (FLC), caspofungin (CFG), micafungin (MFG), anidulafungin (AFG), amphotericin B (AmB), and terbinafine (TRB) were determined *in vitro*. FLC, ITC, VRC, PSC, AmB, and TRB were purchased from Sigma-Aldrich. In addition, ISC was purchased from Targetmol. CFG, MFG, and AFG were purchased from MedChemExpress. A volume of 100 μL of twofold of antifungal agents, as well as the 1:50 diluted inoculum suspension, was added to the microdilution wells using 96-well microtiter plates. The last two columns were growth control wells (containing RPMI 1640 culture medium without antifungal agents) and negative control wells (RPMI 1640 only). The drug concentration ranges (from low to high) were: 0.008–4 mg/L for TRB and AmB, 0.25–128 mg/L for FLC, 0.001–0.5 mg/L for triazoles and 0.25–128 mg/L for echinocandins of *T. marneffei*, 0.008–4 mg/L for echinocandins and 0.25–128 mg/L for triazoles of other *Talaromyces* species. The minimal inhibitory concentration (MIC) was defined as the lowest drug concentration inhibiting 100% of visible growth for triazoles and AmB, 80% for TRB, and a 50% reduction for FLC compared to the drug-free control. The minimal effective concentration (MEC) for echinocandins was determined as the lowest concentration, producing short, stubby, abnormally branched hyphae. Both MIC and MEC were assessed at 48 h. Quality control was maintained using *C. krusei* ATCC 6258 and *C. parapsilosis* ATCC 22019 strains.

### 2.5 Animal models

Species capable of growth at 37°C were evaluated for pathogenic potential using an animal model. Eight-week-old BALB/c mice were immunosuppressed with cyclophosphamide and triamcinolone acetonide as previously described (Zheng et al., 2019). Immunosuppressed mice received an intraperitoneal injection of 100 μL containing 10^7^ conidia. Four weeks post-inoculation, mice were euthanized, and lung tissue was subjected to fungal culture at 27°C and hematoxylin–eosin staining for histopathological examination.

### 2.6 Clinical information

Clinical information was also collected from all 59 patients, including underlying diseases and fungal culture sites.

## 3 Results

### 3.1 Molecular and morphological analysis

Molecular analysis revealed that the 59 isolates belonged to three *Talaromyces* sections: *Talaromyces* section *Talaromyces* (*n* = 50), section *Trachyspermi* (*n* = 8), and section *Islandici* (*n* = 1).

**Figure 1:**
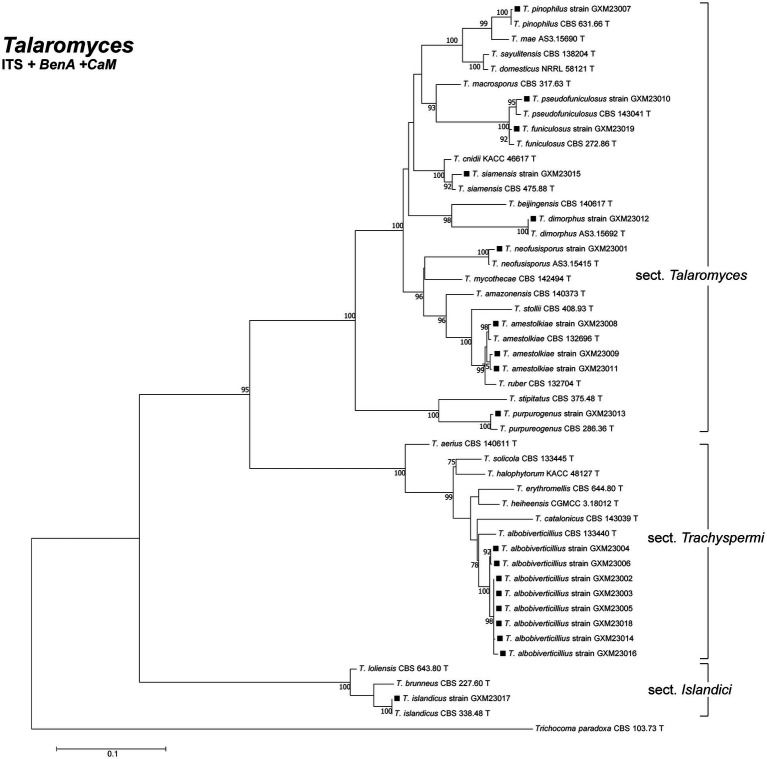
Phylogenetic analysis of clinical *Talaromyces* species in this study. Bootstrap percentages over 70% derived from 1,000 replicates are indicated at the nodes. Strains belonging to this study are indicated in black squares. *Trichocoma paradoxa* (CBS 103633.73 T) was chosen as the outgroup of *Talaromyces*. T: ex-type.

Of the 59 isolates, 40 were identified as *T. marneffei* based on ITS sequence analysis using BLAST in NCBI GenBank and checking in the ISHAM-ITS database (Irinyi et al., 2015). The remaining 19 isolates required phylogenetic analysis incorporating ITS, *BenA*, and *CaM* sequences for species-level identification. Phylogenetic analysis (Figure 1) revealed these 19 isolates to represent 10 species: *T. amestolkiae* (*n* = 3), *T. pinophilus* (*n* = 1), *T. funiculosus* (*n* = 1), *T. pseudofuniculosus* (*n* = 1), *T. neofusisporus* (*n* = 1), *T. siamensis* (*n* = 1), *T. dimorphus* (*n* = 1), *T. purpureogenus* (*n* = 1), *T. albobiverticillius* (*n* = 8), and *T. islandicus* (*n* = 1).

**Figure 2:**
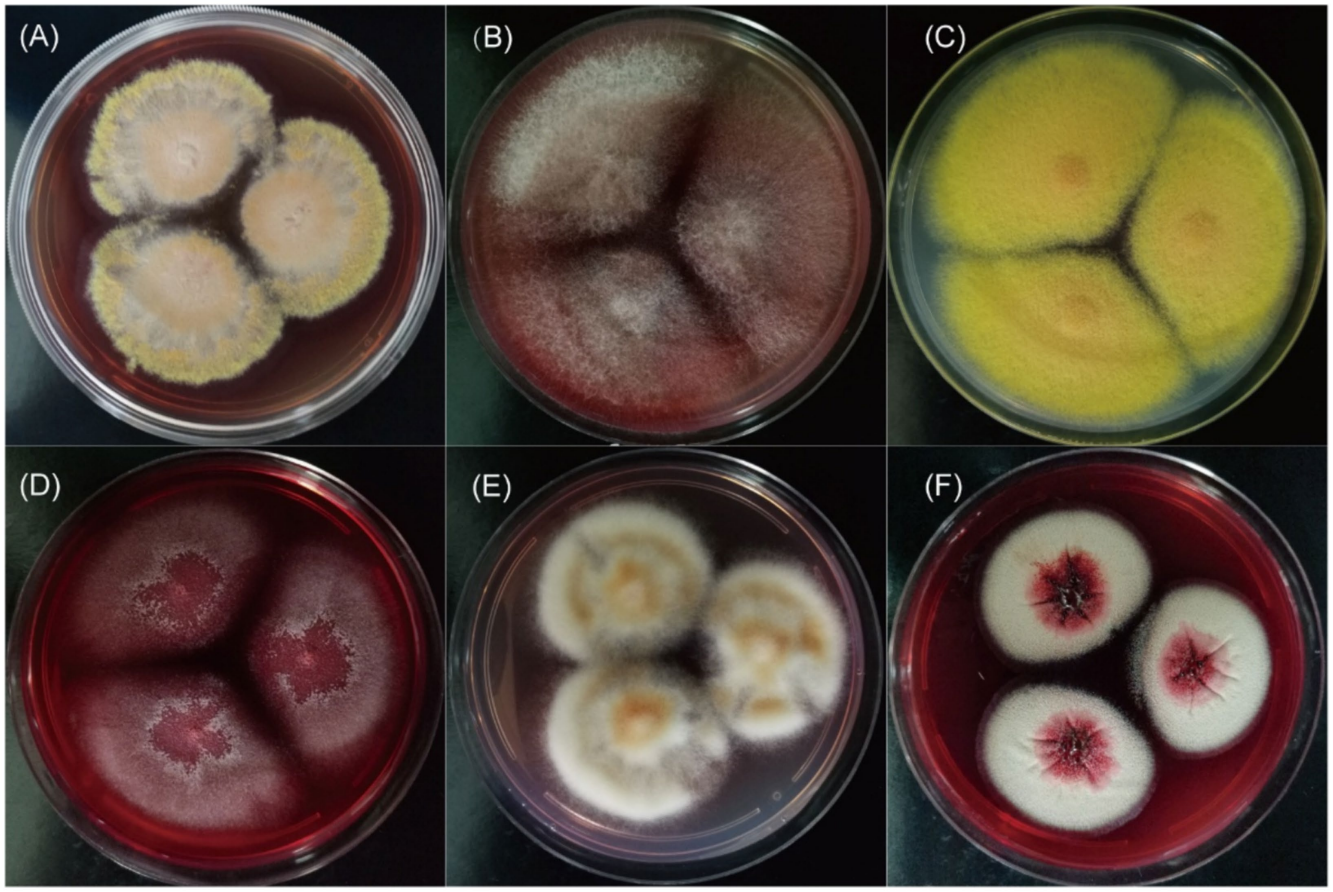
The red pigment of clinical *Talaromyces* species on SDA medium at 25°C for 14 days. **(A–F)** Were *T. marneffei*, *T. amestolkiae*, *T. pinophilus*, *T. neofusisporus*, *T. purpureogenus*, and *T. albobiverticillius*, respectively.

All clinical *Talaromyces* species in this study exhibited yellow-green to grey-green filamentous colonies at 25°C (Supplementary Figure 1). Notably, in addition to *T. marneffei*, five species—*T. amestolkiae*, *T. pinophilus*, *T. neofusisporus*, *T. purpureogenus*, and *T. albobiverticillius*—produced red pigment on SDA medium at room temperature (Figure 2). Interestingly, the microscopic morphology of these red-pigmented species shared similarities with *T. marneffei*, characterized by monoverticillate or biverticillate conidiophores and globose to subglobose conidia (Supplementary Table 1 and Supplementary Figure 3).

Among the species producing abundant red pigment at room temperature, *T. albobiverticillius* (*n* = 8) exhibited limited growth at 37°C. In contrast, *T. amestolkiae*, *T. pinophilus*, *T. siamensis*, *T. islandicus*, *T. neofusisporus*, *T. purpureogenus*, *T. funiculosus*, and *T. marneffei* exhibited growth at 37°C. However, *T. marneffei* was the only species demonstrating thermal dimorphism, adopting a yeast-form growth at 37°C (Supplementary Figure 2).

### 3.2 Antifungal susceptibility testing

Antifungal susceptibility testing was performed on all isolates. The results demonstrated distinct drug susceptibility profiles between *T. marneffei* and the other species (Table 1).

**Table 1:** Results of *in vitro* antifungal susceptibility testing of *Talaromyces* species.

Species (no.)	MIC (mg/L)							MEC (mg/L)		
	FLC	ITC	VRC	PSC	ISC	AmB	TRB	CFG	MFG	AFG
*marneffei* (40)	1–4	0.008–0.03	0.001–0.015	0.001–0.003	0.001–0.015	0.25–2	/	8–16	8–16	8–32
GM	1.83	0.0080	0.0055	0.0018	0.0013	1.09		13.45	11.31	11.12
MIC50	2	0.008	0.008	0.003	0.001	1		16	8	8
MIC90	4	0.015	0.015	0.003	0.003	2		16	16	32
Non-*marneffei* (19)										
*amestolkiae* (3)	>128	>128	32– > 128	>128	64– > 128	0.25–1	0.06–0.125	>4	≤0.008–4	0.5–4
*pinophilus* (1)	>128	>128	32	>128	64	1	0.5	>4	>4	>4
*pseudofuniculosus* (1)	>128	>128	64	>128	>128	1	0.06	>4	>4	4
*funiculosus* (1)	32	8	1	4	4	0.5	0.06	>4	4	4
*neofusisporus* (1)	>128	>128	64	>128	>128	0.125	0.015	4	≤0.008	0.06
*siamensis* (1)	>128	>128	64	>128	>128	0.5	0.25	4	≤0.008	0.125
*dimorphus* (1)	>128	>128	64	>128	4	1	0.125	>4	2	2
*purpureogenus* (1)	>128	>128	16	>128	32	4	2	>4	≤0.008	0.06
*albobiverticillius* (8)	>128	>128	8–64	>128	16– > 128	0.5–2	≤0.008–0.25	2– > 4	≤0.008–0.125	≤0.008–2
*islandicus* (1)	>128	>128	32	>128	64	>4	4	>4	1	>4

Azoles, except for FLC, demonstrated superior activity against *T. marneffei* strains compared to echinocandins and AmB. ITC, VRC, PCS, and ISC exhibited MIC values <0.03 mg/L, while AmB MICs ranged from 0.25 to 2 mg/L. Echinocandins, including CFG, MFG, and AFG, had MEC values ≥8 mg/L.

In contrast to *T. marneffei*, echinocandins displayed superior antifungal activity against other *Talaromyces* species. MEC values of MFG and AFG were ≤0.008–2 mg/L for most strains, while FLC, ITC, and PSC MIC values exceeded 128 mg/L, except for a single *T. funiculosus* isolate.

However, susceptibility to echinocandins varied among strains. *T. amestolkiae*, *T. pinophilus*, *T. funiculosus*, and *T. pseudofuniculosus* (*n* = 1 for each) exhibited high MEC of ≥4 mg/L for all echinocandins. TRB exhibited good antifungal activity against these strains (MIC range ≤0.008–4 mg/L), while AmB showed intermediate activity (MIC range 0.125– > 4 mg/L).

### 3.3 Animal models

Murine models of invasive infection were established to assess the pathogenicity of species capable of growth at 37°C. Survival rates, lung fungal cultures, and histopathological examination of mice were used to evaluate disease progression.

**Figure 3:**
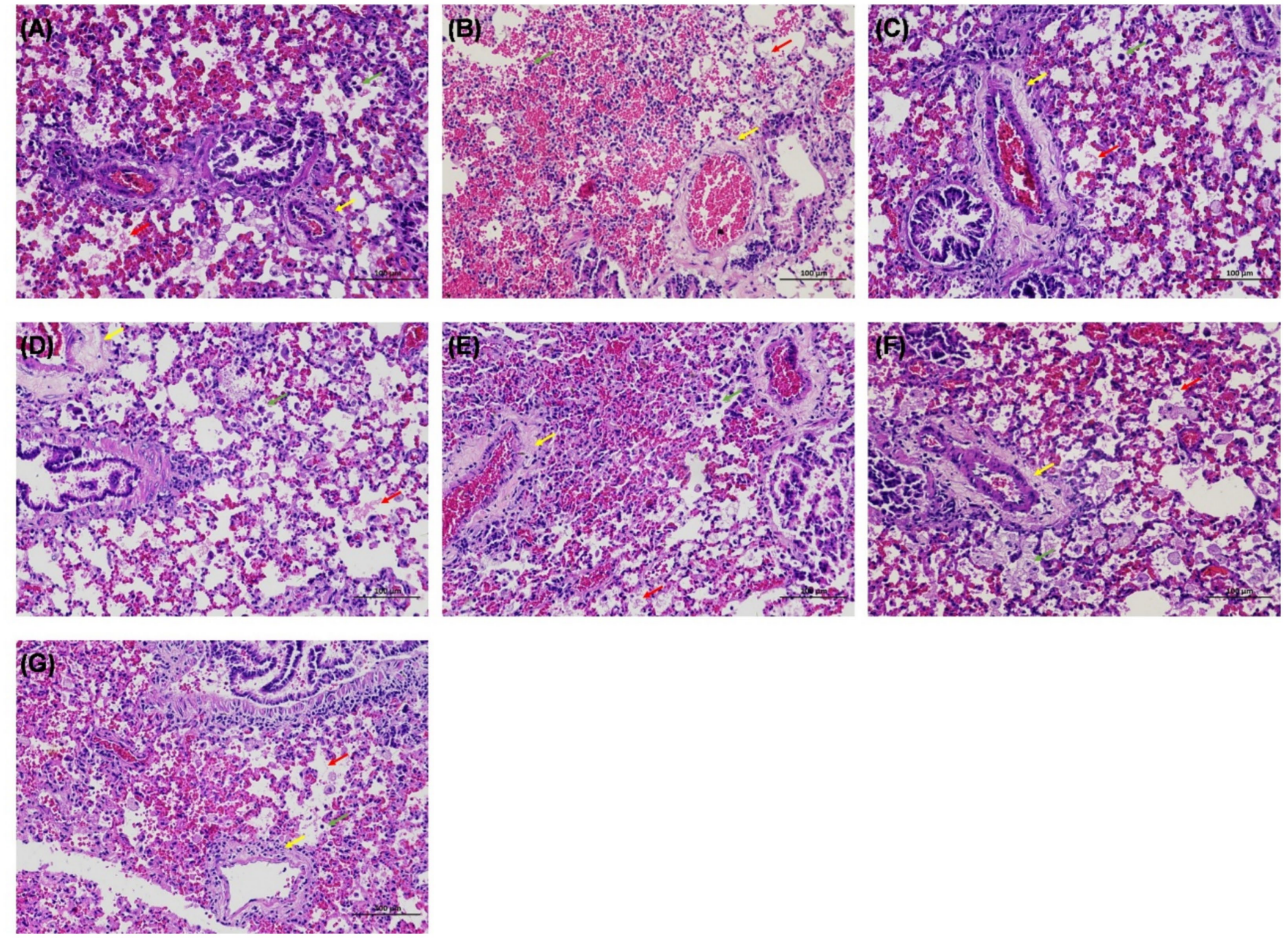
Hematoxylin–eosin (HE) staining of the lung in mice infected with *Talaromyces*. **(A–G)** Were infected by *T. amestolkiae*, *T. pinophilus*, *T. neofusisporus*, *T. funiculosus*, *T. purpureogenus*, *T. siamensis*, and *T. islandicus*, respectively. HE staining showed perivascular edema with lymphocyte infiltration (yellow arrows), and infiltration of eosinophilic flocs (red arrows) and macrophages (green arrows) in the alveolar space.

Except for *T. pinophilus*, all other groups, including *T. amestolkiae*, *T. siamensis*, *T. islandicus*, *T. neofusisporus*, *T. purpureogenus*, and *T. funiculosus*, exhibited mouse mortality within 4 weeks after infection. All infected mice died for the groups of *T. amestolkiae* and *T. funiculosus*, and most for *T. islandicus* (5/ 6), *T. neofusisporus* (5/6), and *T. siamensis* (4/6) infection groups. Histopathological examination of lung tissue from these seven groups showed perivascular edema with lymphocyte infiltration, and infiltration of eosinophilic flocs and macrophages in the alveolar space revealed inflammatory damage (Figure 3). Lung fungal cultures were positive for all mice infected with *T. islandicus* and *T. purpureogenus*, as well as most the mice of *T. pinophilus* (5/6), *T. siamensis* (4/6) *T. funiculosus* (4/6) infection groups (Supplementary Table 2).

### 3.4 Clinical information

The distribution of clinical *Talaromyces* strains across sample types is summarized in Table 2. Among the 11 *Talaromyces* species identified, *T. marneffei* exhibited the broadest range of sample origins, including bronchoalveolar lavage fluid (BALF), sputum, lung tissue, and extrapulmonary specimens such as secretions, skin, lymph nodes, blood, bone marrow, and oral mucosa. In contrast, most (89.5%, 17/19) of *Talaromyces* isolates were recovered from respiratory tract samples (BALF or sputum), with only *T. albobiverticillius* and *T. islandicus* isolated from the skin. Ten strains, primarily *T. albobiverticillius* (*n* = 8), but also including *T. pseudofuniculosus* and *T. dimorphus*, were categorized as environmental contaminants due to their inability to grow at human body temperature (37°C).

**Table 2:** Source of clinical *Talaromyces* strains in this study.

Species (No.)	Source (No.)	Disease of patients (No.)
*T. marneffei* (40)	BALF (20)	AIDS (3), chronic obstructive pulmonary disease (1), pneumonia (2), lung cancer (5), pulmonary tuberculosis (1), septicemia (2), systemic lupus erythematosus (1), anti-IFN-γ autoantibodies positive (5)
	Secretions (8)	Systemic sclerosis (1), anti-IFN-γ autoantibodies positive (6), multiple organ dysfunction syndrome (1)
	Skin (4)	Multiple organ dysfunction syndrome (1), anti-IFN-*γ* autoantibodies positive (3)
	Lymph nodes (2)	Anti-IFN-γ autoantibodies positive (2)
	Lung tissue (2)	AIDS (1), pulmonary tuberculosis (1)
	Sputum (1)	Lung cancer
	Blood (1)	Nephrotic syndrome
	Bone marrow (1)	Primary immunodeficiency disease (1)
	Oral mucosa (1)	Hydroa vacciniforme-like lymphoma (1)
*T. amestolkiae* (3)	Sputum (2)	Lung cancer (1), unknown (1)
	BALF (1)	Tuberculous pleuritis (1)
*T. pinophilus* (1)	Sputum (1)	Kartagener syndrome (1)
*T. pseudofuniculosus* (1)	BALF (1)	Sjogren syndrome (1)
*T. funiculosus* (1)	BALF (1)	Pneumonia (1)
*T. neofusisporus* (1)	BALF (1)	Anti-IFN-γ autoantibodies positive (1)
*T. siamensis* (1)	BALF (1)	Lung cancer (1)
*T. dimorphus* (1)	BALF (1)	Pneumonia (1)
*T. purpureogenus* (1)	BALF (1)	Chronic cough (1)
*T. albobiverticillius* (8)	Skin (1)	Unknown (1)
	BALF (7)	Acute myeloid leukemia (1), T lymphoblastic lymphoma (1), hemophagocytic syndrome (1), lung cancer (1), chronic obstructive pulmonary disease (2), pneumonia (1)
*T. islandicus* (1)	Skin (1)	Unknown (1)

Of the 49 patients whose isolates grew at 37°C, 95.9% (47/49) had underlying diseases. The distribution of underlying diseases differed between patients infected with *T. marneffei* and those infected with other *Talaromyces* species. Among the 40 patients infected with *T. marneffei*, 40% were positive for anti-IFN-*γ* autoantibodies. In contrast, respiratory diseases were the primary underlying condition in patients infected with other *Talaromyces* species.

## 4 Discussion

Emerging human pathogenic fungi that were previously thought to be environmental could be driven by warming (Casadevall, 2023). Southern China’s warm and humid climate fosters a diverse fungal environment, making it a hotspot for fungal infections, including talaromycosis caused by *T. marneffei* (Hu et al., 2013; Cao et al., 2019). Although other *Talaromyces* species are commonly reported in the natural environment, several clinical cases have shown their potential pathogenicity. While *T. marneffei* is prevalent, clinical cases involving other *Talaromyces* species are underreported in this region. Our study identified 11 *Talaromyces* species from three distinct sections among clinical samples: *T. amestolkiae*, *T. pinophilus*, *T. funiculosus*, *T. pseudofuniculosus*, *T. neofusisporus*, *T. siamensis*, *T. dimorphus*, *T. purpureogenus*, *T. albobiverticillius*, *T. islandicus*, and *T. marneffei*. Although *T. marneffei* was the most common, other species constituted 32.2% of the isolates, demonstrating the region’s rich *Talaromyces* diversity in a clinical setting.

Red pigment production at room temperature is a common characteristic among *Talaromyces* species, including *T. purpureogenus*, *T. albobiverticillius*, *T. minioluteus*, and *T. atroroseus* (Yilmaz et al., 2012; Frisvad et al., 2013). Relying solely on gross morphology and red pigment production at 25°C for species identification can lead to the misidentification of these species as *T. marneffei*. In this study, we observed red pigment production in several clinical *Talaromyces* species other than *T. marneffei*, specifically *T. amestolkiae*, *T. pinophilus*, *T. neofusisporus*, *T. purpureogenus*, and *T. albobiverticillius*. Consequently, heightened attention should be given to these species, particularly in the context of *T. marneffei* research. To differentiate these species from *T. marneffei*, cultivating fungal isolates at both 25°C and 37°C is crucial, as *T. marneffei* exhibits yeast-form growth at 37°C.

Understanding antifungal drug susceptibility is crucial for guiding the clinical management of fungal infections. Previous studies have demonstrated significant differences in drug susceptibility profiles between *T. marneffei* and other *Talaromyces* species (Liu et al., 2013; Guevara-Suarez et al., 2016; Lau et al., 2017; Guo et al., 2021). Consistent with these findings, our study revealed high triazole susceptibility but strong echinocandin resistance in *T. marneffei* strains. Conversely, most other *Talaromyces* species exhibited high susceptibility to echinocandins, moderate susceptibility to AmB and TRB, and lower susceptibility to azoles. Notably, the antifungal susceptibility for echinocandins of some strains in our study was low, probably due to the potential variability in antifungal susceptibility among different *Talaromyces* species and geographic regions. Therefore, antifungal susceptibility testing of clinical *Talaromyces* strains is necessary for appropriate antifungal treatment to avoid toxic side effects caused by unnecessary drug exposure. However, due to the limitations of the sample of this study, it is expected that more in-depth research will be conducted in the future to verify this view. Consequently, tailoring antifungal treatment based on drug susceptibility testing is recommended. In the absence of timely susceptibility data, TRB, AmB, MFG and AFG among the echinocandins may be considered as initial empiric therapy.

Clinically, unlike the diverse sources of *T. marneffei* isolates, other *Talaromyces* species are predominantly recovered from respiratory specimens. In this study, a substantial proportion (89.5%, 17/19) of *Talaromyces* strains originated from BALF or sputum. This finding aligns with the growing body of evidence implicating *Talaromyces* species in respiratory tract infections. For instance, *T. amestolkiae* has been isolated from BALF in both an AIDS patient and an immunocompetent female with pneumonia (Yilmaz et al., 2012). Additionally, *T. pinophilus*, *T. funiculosus*, *T. purpureogenus*, *T. albobiverticillius*, and *T. islandicus* have been reported in human respiratory tract infections (Yilmaz et al., 2012; Guevara-Suarez et al., 2016; Li et al., 2019; Guo et al., 2021). Generally, the capacity to thrive at human body temperature is a prerequisite for fungal infections in humans. Our study found that patients infected with *Talaromyces* species capable of growth at 37°C predominantly exhibited underlying conditions associated with immunosuppression, such as lung cancer, Kartagener syndrome, and anti-IFN-*γ* autoantibodies. These findings align with previous research demonstrating a correlation between immunocompromised states and infections caused by these fungi (Atalay et al., 2016; Villanueva-Lozano et al., 2017; Guo et al., 2021). These results suggest a predilection for respiratory tract infections and a reliance on a host with a compromised immune system for successful colonization. And they indirectly indicate that there are similarities in the pathogenesis of clinical *Talaromyces* species in different regions. However, whether the same species in different regions show this trend needs to be explored in the future. To explore the potential pathogenicity and validate Koch’s postulates experimentally, we established a murine infection model using immunosuppressed hosts, and clinical *Talaromyces* strains re-isolated from diseased mice were successfully. The observed lung inflammation in mice infected with these *Talaromyces* species further supports this hypothesis. Notably, in several species, the mortality in mice did not agree with the lung fungal cultures due to the low sensitivity of fungal cultures and the low toxicity of some species.

Our study has certain limitations that should be acknowledged. Due to institutional constraints regarding specialized instrumentation and heightened biosafety considerations, we use intraperitoneal injection to study pulmonary infection in murine models. Notably, in *T. marneffei* infection models, intraperitoneal injection has been widely adopted and validated (Zheng et al., 2019; He et al., 2022). These investigations demonstrated pulmonary involvement with fungal culture or histopathological evidence despite extra-respiratory inoculation by intraperitoneal injection. The respiratory tract infection models of these emerging *Talaromyces* species challenge as a future research direction. And secondly, it is the small sample size derived from a single center. To enhance the generalizability of our findings and potentially identify additional *Talaromyces* pathogen species, future studies should involve larger sample sizes collected from multiple centers.

In conclusion, Southern China exhibits a diverse clinical spectrum of *Talaromyces* species, some of which can be easily misidentified as *T. marneffei*. Certain *Talaromyces* species can grow at 37°C and have demonstrated pathogenicity in animal models. Since *T. marneffei* and other *Talaromyces* species require different drug treatments, misdiagnosis can result in inappropriate therapy and treatment failure. Consequently, when *Talaromyces* strains are isolated from respiratory tract specimens of immunocompromised individuals, clinicians should consider their potential pathogenicity and initiate appropriate treatment.

## Data Availability

The sequences used for phylogenetic analysis in this study have been deposited at NCBI GenBank. The accession numbers were as follows: OQ550077-OQ550095 and PQ37528-PQ37567 for ITS region, PP869295-PP869313 and PQ756998-PQ757037 for *BenA*, PQ095599-PQ095617 and PQ661880-PQ661919 for *CaM*.
