# Supplementary material for: Exploring the diversity and pathogenicity of Talaromyces species isolated from clinical in Southern China
Source: Front Microbiol. 2025 Jun 26;16:1610481. doi: 10.3389/fmicb.2025.1610481 (PMC12241079; doi:10.3389/fmicb.2025.1610481)
Supplement: Supplementary file 1 [file Data_Sheet_1.docx]

Supplementary Material

# Supplementary Tables

**Supplementary Table 1.** **Morphological characterization of clinical *Talaromyces* species.**

| Species name |  | Growth at 25°C | | Growth at 37°C | Microscopic structure | |
| --- | --- | --- | --- | --- | --- | --- |
|  |  | Colony | soluble red pigment |  | Conidiophore branching | Conidial shape |
| *T. marneffei* | SDA | Velvety yellow-green colony | Large amount | Yeast phase and divide by fission | Mono- to biverticillate | Subglobose |
|  | MEA | Velvety yellow-green colony | Large amount | Yeast phase and divide by fission |  |  |
| *T. amestolkiae* | SDA | Loosely yellow-green colony | Large amount | Yellow-green filamentous colony | Mono- to Biverticillate | Ellipsoidal |
|  | MEA | Velvety grey-green colony | Absent | Yellow-green filamentous colony |  |  |
| *T. pinophilus* | SDA | Loosely yellow to yellow-green colony | Very weak | Yellow-green to grey-green filamentous colony | Biverticillate | Globose to subglobose |
|  | MEA | Velvety grey-green colony | Absent | Grey-green filamentous colony |  |  |
| *T. funiculosus* | SDA | Loosely orange-red to yellow-green colony | Absent | Grey-green filamentous colony | Biverticillate | Ellipsoidal |
|  | MEA | Velvety grey-green colony | Absent | Grey-green filamentous colony |  |  |
| *T. pseudofuniculosus* | SDA | Velvety grey-green colony | Absent | No growth | Biverticillate | Ellipsoidal |
|  | MEA | Velvety grey-green colony | Absent | No growth |  |  |
| *T. neofusisporus* | SDA | Loosely white to yellow-green colony | Large amount | Grey-green filamentous colony | Biverticillate | Subglobose |
|  | MEA | Velvety grey-green colony | Absent | Grey-green filamentous colony |  |  |
| *T. siamensis* | SDA | Velvety white to yellow-green colony | Absent | Yellow-green to grey-green filamentous colony | Biverticillate | Ellipsoidal |
|  | MEA | Velvety grey-green colony | Large amount | Grey-green filamentous colony |  |  |
| *T. dimorphus* | SDA | Loosely white to yellow-green colony | Absent | No growth | Mono- to Biverticillate | Ellipsoidal |
|  | MEA | Velvety grey-green colony | Absent | No growth |  |  |
| *T. purpureogenus* | SDA | Loosely white to yellow-green colony | Large amount | Yellow-green filamentous colony | Biverticillate | Subglobose |
|  | MEA | Loosely white to yellow-green colony | Absent | Grey-green filamentous colony |  |  |
| *T. albobiverticillius* | SDA | Velvety white to grey-green colony | Large amount | No growth | Biverticillate | Globose to subglobose |
|  | MEA | Velvety yellow-green to grey-green colony | Absent | No growth |  |  |
| *T. islandicus* | SDA | Loosely white to brownish colony | Absent | Grey-green filamentous colony | Biverticillate | Ellipsoidal |
|  | MEA | Velvety grey-green colony | Absent | Grey-green filamentous colony |  |  |

**Supplementary Table 2. Results of murine models of invasive *Talaromyces* infection**

| species | Death of mice | Time of death (days after infection/numbers) | Positive of Lung fungal culture |
| --- | --- | --- | --- |
| *T. amestolkiae* | 6/6 | +3(1), +4(3), +5(1), +6(1) | 2/6 |
| *T. pinophilus* | 0/6 | / | 5/6 |
| *T. siamensis* | 4/6 | +4(1), +9(1), +11(1), +12(1) | 4/6 |
| *T. islandicus* | 5/6 | +2(1), +4(3), +5(1) | 6/6 |
| *T. neofusisporus* | 5/6 | +3(2), +5(2), +10(1) | 2/6 |
| *T. purpureogenus* | 3/6 | +4(2), +10(1) | 6/6 |
| *T. funiculosus* | 6/6 | +3(4), +4(1), +5(1) | 4/6 |

**Supplementary Table 3. Clinical manifestations of *Talaromyces* infection in this study**

| Species (No.) | Clinical Symptoms (No.) | | | | Antifungal Treatment and Outcomes (No.) |
| --- | --- | --- | --- | --- | --- |
|  | Fever | Cough | Bone destruction | Skin lesions |  |
| *T. marneffei* (40) | 15 | 24 | 8 | 10 | Clinical improvement following AmB (5), VRC (18), ITC (7), AmB + VRC (2), VRC+ITC (1);  Suboptimal efficacy of AmB (2), VRC (2), VOC + AmB (2);  Didn’t receive antifungal therapy (1) |
| *T. amestolkiae* (3) | 2 | 2 |  |  | Didn’t receive antifungal therapy (2)  Clinical improvement following VRC (1). |
| *T. pinophilus* (1) |  | 1 |  |  | Didn’t receive antifungal therapy (1) |
| *T. funiculosus* (1) |  | 1 |  |  | Didn’t receive antifungal therapy (1) |
| *T. neofusisporus* (1) | 1 | 1 | 1 |  | Suboptimal efficacy of ITC (1) |
| *T. siamensis* (1) |  | 1 |  |  | Didn’t receive antifungal therapy (1) |
| *T. purpureogenus* (1) | 1 |  |  |  | Didn’t receive antifungal therapy (1) |
| *T. islandicus* (1) |  |  |  | 1 | Didn’t receive antifungal therapy (1) |

#

# Supplementary Figures


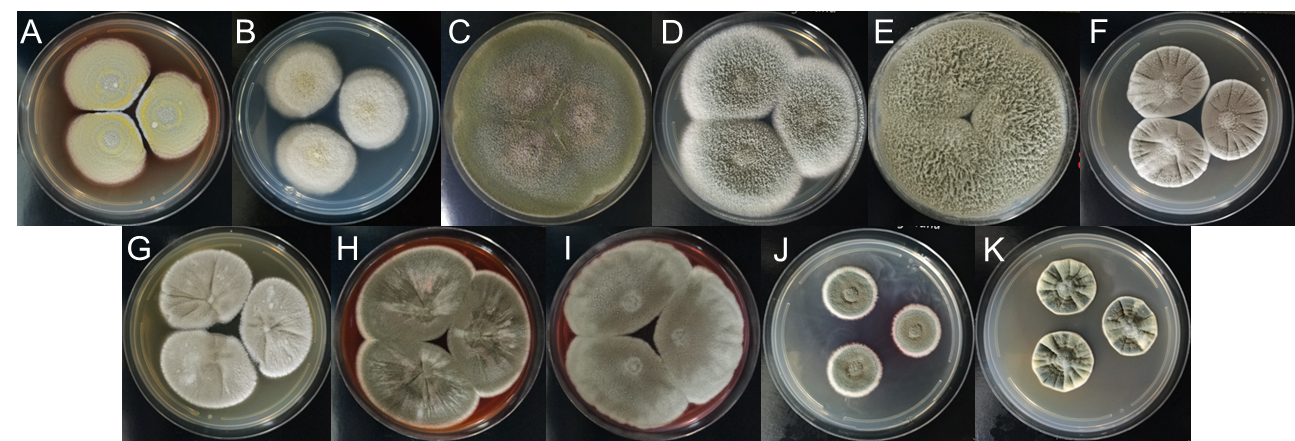


**Supplementary Figure 1.** ***Talaromyces* species in this study grow on Czapek Yeast Autolysate Agar (CYA) medium at 25℃ for 14 days.** A-K were *T. marneffei*, *T. amestolkiae*, *T. pinophilus*, *T. funiculosus*, *T. pseudofuniculosus*, *T. neofusisporus*, *T. dimorphus*, *T. siamensis*, *T. purpureogenus*, *T. albobiverticillius*, and *T. islandicus*, respectively.


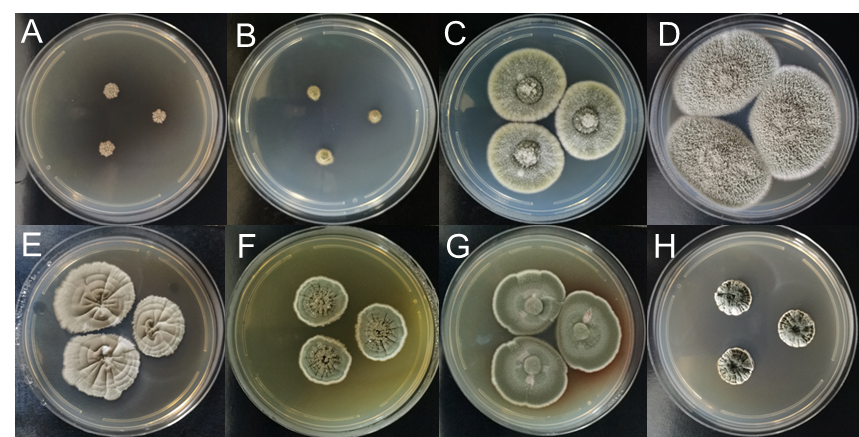


**Supplementary Figure 2.** ***Talaromyces* species in this study grow on CYA medium at** **37℃ for 14 days.** A-H were *T. marneffei*, *T. amestolkiae*, *T. pinophilus*, *T. funiculosus*, *T. neofusisporus*, *T. siamensis*, *T. purpureogenus*, and *T. islandicus*, respectively.


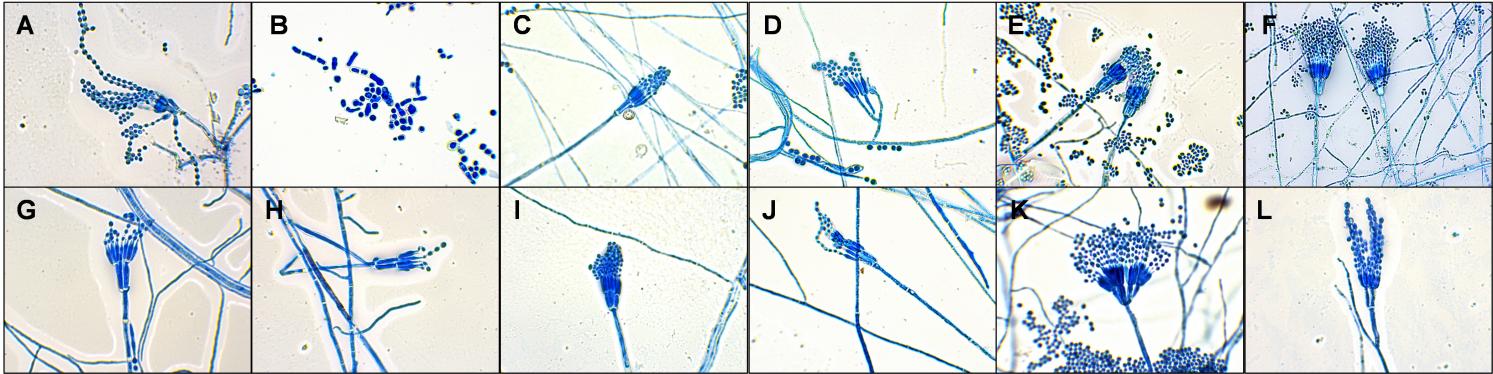


**Supplementary Figure 3.** **Microscopic features of *Talaromyces* species in this study (1000 ×).** A-L were *T. marneffei* 25℃, *T. marneffei* 37℃, *T. amestolkiae*, *T. pinophilus*, *T. funiculosus*, *T. pseudofuniculosus*, *T. neofusisporus*, *T. dimorphus*, *T. siamensis*, *T. purpureogenus*, *T. albobiverticillius*, and *T. islandicus*, respectively.
